# Severity and management of psoriasis within primary care

**DOI:** 10.1186/s12875-016-0544-6

**Published:** 2016-10-14

**Authors:** Alan G. Wade, Gordon M. Crawford, David Young, Joyce Leman, Neil Pumford

**Affiliations:** 1 Patients Direct, 3 Todd Campus, West of Scotland Science Park, Glasgow, G20 0XA UK; 2 Department of Mathematics and Statistics, University of Strathclyde, Glasgow, G1 1XQ UK; 3 Western Infirmary, Glasgow, G11 6NT UK; 4 AbbVie Ltd, Abbott House, Vanwall Business Park, Vanwall Road, Maidenhead, SL6 4XE UK

**Keywords:** Psoriasis, Psoriatic arthritis, Primary health care, General practice, Survey, SIGN, DLQI, SAPASI, Comorbidity

## Abstract

**Background:**

Scottish Intercollegiate Guidelines Network and National Institute of Health and Care Excellence guidelines stress the importance of assessing patients with psoriasis for psoriatic arthritis, comorbidities associated with severe disease and quality of life (QoL).

The purpose of the study was to evaluate the primary care management of psoriasis in relation to disease severity and QoL from a patient’s perspective.

**Methods:**

A cross-sectional survey of adults (≥18 years) with psoriasis managed in primary care was conducted in Scotland over 1-year (2012–2013). Patients with psoriasis were identified and invited to participate in the online/telephone survey. The questionnaires included; Dermatology Life Quality Index (DLQI), Self-Administered Psoriasis Area and Severity Index (SAPASI), Psoriasis Epidemiology Screening Tool (PEST). The primary outcome measure was DLQI. Secondary outcomes included; demographics; comorbidities; involvement of different body sites; SAPASI and PEST scores. Relationships between measures were analysed using univariate analysis.

**Results:**

The mean age of patients (*n* = 905) was 54.5 years (SD = 16.1), 436 (48.2 %) were men, and median DLQI and SAPASI scores were 4.0 and 6.0, respectively. Current psoriasis treatments were topical only (587, 64.9 %), oral medications or phototherapy (122, 13.5 %), biologics (26, 3 %) and none (156, 17.2 %). Despite SIGN recommendations, 256 of 391 patients (65.5 %) with a DLQI >5 (at least a moderate effect on QoL) had not seen a specialist during the past year. According to PEST scores, 259 patients (28.6 %) had symptoms suggestive of psoriatic arthritis requiring rheumatology referral.

**Conclusion:**

National recommendations are not being fully implemented in primary care in patients with psoriasis or psoriatic arthritis.

## Background

Psoriasis is a common, chronic inflammatory skin disease, which affects 1 to 2 % of the population in the United Kingdom (UK). The disease can vary widely in severity; many people with psoriasis have an impaired quality of life (QoL) due to the significant impact of the disease on functional, psychological, and social well-being.

Several different body sites can be affected, including areas that are difficult to conceal, such as the face, scalp, and nails. The presence of visible lesions can have a particularly marked impact on a patient’s QoL [1]. In the UK, the patient-reported Dermatology Life Quality Index (DLQI) is the most commonly used measure of QoL in patients with psoriasis [2]. It is a simple, validated tool consisting of 10 questions, is easy to use in primary care, and allows measurement of limited disease, which may be associated with significant psychosocial impact.

The importance of assessing all patients with psoriasis for psoriatic arthritis and comorbidities associated with severe disease, such as diabetes mellitus, dyslipidaemia, hypertension, coronary heart disease, and depression, is stressed in both the Scottish Intercollegiate Guidelines Network (SIGN) and the National Institute for Health and Care Excellence (NICE) guidelines [3, 4]. Obesity and lifestyle factors, such as smoking and excessive alcohol intake, may also be associated with psoriasis and could affect other comorbidities [5].

As the present survey was conducted in the West of Scotland, the SIGN guidelines for the management of psoriasis within primary care are relevant to this patient population. These guidelines recommend that patients with psoriasis have annual visits for DLQI score assessment and screening for articular symptoms and comorbidities. They also advise that patients with suspected psoriatic arthritis should be referred to a rheumatologist for assessment.

The primary objective of this study was to evaluate the distribution of DLQI scores amongst patients with psoriasis within primary care, thus providing a cross-sectional insight into the impact of the disease, and to correlate disease impact with the management of psoriasis. Secondary objectives were to compare the patient Self-Administered Psoriasis Area and Severity Index (SAPASI) with treatments, comorbidities, and DLQI scores, and to evaluate the prevalence of comorbidities and the involvement of scalp, nails, palms or soles, and joints.

## Methods

### Selection and description of participants

Patients in a primary care setting were recruited for this survey. General practitioners (GPs) within the West of Scotland were contacted and all practices willing to participate were included. At each practice, all adult patients (aged ≥18 years) with psoriasis were identified using patient codes for psoriasis. Patients with other unspecified skin conditions and/or receiving specified therapies for psoriasis (e.g., tar products and systemic therapies such as methotrexate and acitretin) were also identified and were eligible for study inclusion if a diagnosis of psoriasis was validated by their GP. All eligible patients were invited to participate. Informed consent was obtained from participants for completion of the survey online or by telephone and for their medical records to be evaluated for additional information. No selection criteria were applied at the stage of practice or patient selection that could introduce bias. The National Research Ethics Centre confirmed that formal ethical approval was not required.

### Data collection

#### Patient survey

The study was conducted over a 1-year period between December 2012 and December 2013. The survey collected demographic details and information on comorbidities, alcohol and smoking intake, disability living allowance (DLA) and/or work status, and the involvement of different sites such as nails, scalp, and joints. The DLQI was the main assessment tool used in this study. However, the SAPASI, a patient-assessed version of the widely used Psoriasis Area and Severity Index (PASI) designed to measure the severity of psoriasis, and the Psoriasis Epidemiology Screening Tool (PEST), designed to screen for psoriatic arthritis, were also included [6, 7]. Patients completed the questionnaire online or used a free-of-charge telephone line and were led through the same questionnaire by a nurse who had been trained on clinical trials, the disease area, and the specific project.

#### Sample size and statistical methods

As the project was exploratory, no formal sample size calculations were conducted. Patient characteristics, DLQI and SAPASI scores, and responses were summarised descriptively. Data were summarised as the number of individuals for whom data were available and the number with missing data for each variable. Associations between outcomes (such as DLQI and affected site) were determined using univariate analysis (chi-square tests for trend). The Mann–Whitney test was used to compare quantitative variables between groups and the Pearson correlation coefficient was used to quantify relationships between variables. Statistical analyses were performed using Minitab, version 16 (Pennsylvania State University, PA, USA) on the full analysis set, with no imputation of missing data.

## Results

Twenty-seven practices in the West of Scotland agreed to participate in the study. A total of 990 patients began the survey; 905 (91.5 %) completed it. GP reports were obtained for 688 of these patients (76.0 %).

### Demographic data

The mean age of participating patients was 54.5 years; similar numbers of men and women participated in the survey (Table 1). The majority of patients (743; 82.1 %) reported that they were receiving no treatment (*n* = 156) or topical treatment only (*n* = 587) for psoriasis (Table 1).

**Table 1:** Demographic and Disease Characteristics, Patient Survey Data

Characteristic	(*n* = 905)
Sex, *n* (%)	
Male/female	436/469 (48.2/51.8)
Age	
Mean (SD)	54.53 (16.07)
Patient care, n (%)	
Primary care only (general practitioner)	688 (76.0)
Specialist^a^	217 (24.0)
DLQI	
Mean (SD)	6.143 (6.198)
Median	4.0
Range	0–29
DLQI category: effect on patient’s life, *n* (%)	
No effect (0–1)	249 (27.5)
Small effect (2–5)	265 (29.3)
Moderate effect (6–10)	204 (22.5)
Very large effect (11–20)	150 (16.6)
Extremely large effect (21–30)	37 (4.1)
SAPASI	
Mean (SD)	6.369 (5.612)
Median	6.00
Range	0–59.76
Areas involved, *n* (%)	
Limbs	669 (73.9)
Scalp	563 (62.2)
Trunk	432 (47.7)
Hands	324 (35.8)
Feet	247 (27.3)
Face	247 (27.3)
Nails	288 (31.8)
Genital area	167 (18.5)
Plaques on palms of hands	112 (12.4)
Plaques on soles of feet	101 (11.2)
Current treatments for psoriasis, *n* (%)	
No treatment	156 (17.2)
Topical treatment only^b^	587 (64.9)
Oral drugs	68 (7.5)
Methotrexate	31 (3.4)
Phototherapy	54 (6.0)
Biologics	26 (2.9)
Other treatments (non-prescribed)	29 (3.2)
Missing	14 (1.5)
Presence of comorbidities, *n* (%)	
Cardiovascular disease (such as angina, heart problems, high blood pressure)	247 (27.3)
High cholesterol	158 (17.5)
Depression/anxiety problems	152 (16.8)
Chest/respiratory problems (eg, asthma, bronchitis, COPD)	111 (12.3)
Diabetes	81 (9.0)
Obesity^c^	77 (8.5)
Cerebrovascular disease (such as stroke, haemorrhage, aneurysm)	28 (3.1)
Cancer (excluding skin cancer)	25 (2.8)
Liver disease	9 (1.0)

*DLQI* Dermatology Life Quality Index, *SAPASI* Self-Administered Psoriasis Area and Severity Index, *SD* standard deviation, *COPD* chronic obstructive pulmonary disease

^a^Includes patients seen by a dermatologist, dermatologic nurse, and those receiving oral or injection therapy

^b^A total of 707 patients received topical treatment; 120 patients received topical and other treatment

^c^Weight and height data were not collected; thus, self-reported obesity could not be verified and is likely to have been underreported by participants

#### Impact of disease (DLQI)

**Fig. 1:**
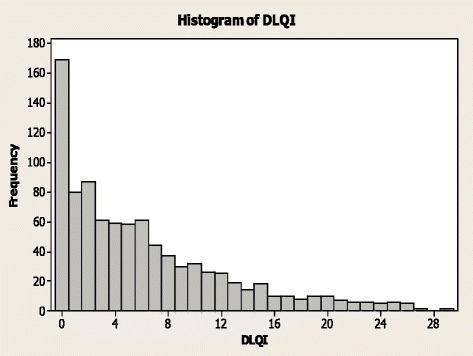
DLQI Scores, Patient Survey Data. *DLQI* Dermatology Life Quality Index

Patient DLQI scores are shown in Fig. 1. Psoriasis had only a small or no effect on QoL (DLQI ≤5) in more than half of the patients (514; 56.8 %). However, for 391 patients (43.2 %), the condition had at least a moderate effect on QoL (DLQI >5; Table 1).

Of the 391 patients with a DLQI >5, 135 (34.5 %) had seen a specialist in the past year and 256 (65.5 %) had not been seen in secondary care. Although 156 patients were receiving no treatment for their psoriasis, 28 (17.9 %) had psoriasis that was affecting their lives to at least a moderate effect (DLQI >5), indicating an unmet need for treatment.

Involvement of different sites, such as nails, scalp, palms, and soles, are presented in Table 1. In univariate analyses, there were significant associations between DLQI category (0-5, 6-10, and 11-30) and affected site for face, scalp, trunk, limbs, genitals, hands, feet, and nails. Patients with psoriasis present at these sites were more likely to be in a higher DLQI category. For example, 51.2, 78.1, and 62.2 % of those in DLQI categories 0-5, 6-10, and 11-30 had scalp involvement compared with 48.9, 21.9, and 24.5 % with no scalp involvement for the respective categories (chi-square for trend = 54.513; *P* < 0.001). However, the results are difficult to interpret, as patients generally have disease involvement at multiple sites.

### Severity of disease (SAPASI)

**Fig. 2:**
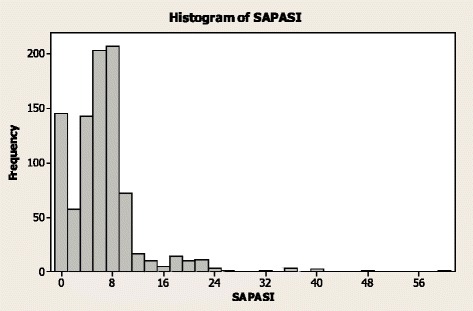
SAPASI Scores, Patient Survey Data. *SAPASI* Self-Administered Psoriasis Area and Severity Index

**Fig. 3:**
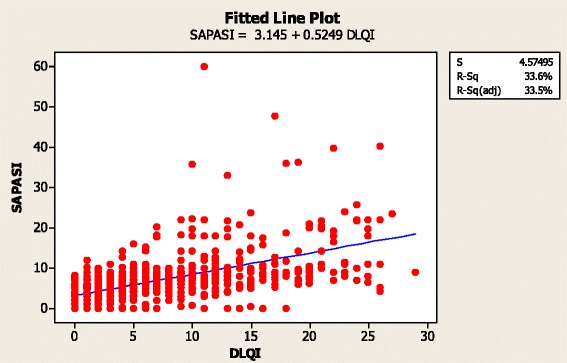
Relationship Between DLQI and SAPASI Scores, Patient Survey Data. *DLQI* Dermatology Life Quality Index, *SAPASI* Self-Administered Psoriasis Area and Severity Index

The SAPASI scores are shown in Fig. 2. There was a significant correlation between patient-reported DLQI and SAPASI scores (*r* = 0.580; *P* < 0.001), although there was considerable variability (Fig. 3).

### PEST questionnaire

On the PEST questionnaire, 259 respondents (28.6 %) had a total of ≥3 positive responses, which signified possible psoriatic arthritis, and indicated that a referral to a rheumatologist was required. Both patient-reported and GP medical record data noted that a smaller proportion of patients had been diagnosed with psoriatic arthritis than indicated from the PEST scale results. Ninety-six (11.9 %) of the 809 patients with data self-reported that they had received a diagnosis of psoriatic arthritis and 70 (13.8 %) of the 506 patients with GP medical record data had a diagnosis of psoriatic arthritis reported by the GP.

We report both the results of the GP survey and also that of a more intensive scrutiny of GP notes, where the diagnosis of psoriatic arthritis was reported by the patient, but not in the initial GP survey. There was no agreement between patients and GPs in the reporting of diagnoses of psoriatic arthritis (κ = −0.276). The poor correlation between the patient and GP reports of the diagnosis of psoriatic arthritis was surprising and led to further investigation.

The data of 96 patients who had reported a diagnosis of psoriatic arthritis that was not confirmed in the original GP survey were stringently reviewed (Table 2).

**Table 2:** Patient-Reported Psoriatic Arthritis: HCP Reported as Having Made Diagnosis and Percentage Recorded in GP Survey

HCP	*n*	Notes reviewed	Positive record in GP notes	No record in GP notes
GP	24	22	8 (36 %)	14 (64 %)
Dermatologist	12	11	8 (73 %)	3 (27 %)
Rheumatologist	55	45	43 (96 %)	2 (4 %)
Other	5	5	1 (20 %)	4 (80 %)
Total	96	83	60 (72 %)	23 (28 %)

*GP* general practitioner, *HCP* healthcare provider

Of the 96 records, 83 could be reviewed; 60 of these patients (72 %) had a record of a diagnosis of psoriatic arthritis that had not been reported by the GP in the original survey. When the patient reported that the diagnosis had been by a hospital specialist, this appeared to have a much closer correlation with the diagnosis being recorded in the notes (96 % of patients reporting a rheumatologist and 73 % of patients reporting a dermatologist made the original diagnosis). All but two records where the patient reported the diagnosis having been made by a rheumatologist in fact contained the information. For other sources of diagnosis, the record appeared less robust (36 % of GPs and 20 % other).

Seventy-two percent of the records reviewed more rigorously had evidence that the diagnosis was correctly reported by the patient, indicating that it was not easily accessible or determined by the GPs originally completing the survey.

There still remains a potential issue in the identification of psoriatic arthritis; 109 patients with a PEST scale score ≥3 did not have a recorded diagnosis of psoriatic arthritis, which may be due to the absence of a referral to a rheumatologist and a lack of a definitive diagnosis rather than a recording in the GP records of “arthritis or joint pains”.

### Presence of comorbidities

Cardiovascular disease was the most commonly reported comorbidity (Table 1). In univariate analyses, the diagnosis of depression/anxiety as a comorbidity was associated with a higher DLQI than expected (chi-square for trend = 13.307; *P* <0.001).

### Lifestyle factors

Lifestyle factors (alcohol/smoking) and the number of patients on DLA and having time off work because of psoriasis are shown in Table 3. Thirty-seven patients (8 % of those working) reported having taken some time off work because of their disease in the previous 30 days and had significantly higher median DLQI scores than those who had not taken any time off (12 vs 5, Mann–Whitney test; *P* < 0.001).

**Table 3:** Smoking, Alcohol Intake, Employment Status, Time Off Work Because of Psoriasis, and DLQI Scores, Patient Survey Data

	*N* (%) of patients (*n* = 905)
Smoking status, *n* (%)	
Smoker	212 (23.4)
Non-smoker	385 (42.5)
Ex-smoker	308 (34.0)
Alcohol weekly intake (units)	
Mean (SD)	7.2 (12.3)
Median	3.0
Range	0–200
Employment/education status, *n* (%)	
No – not currently employed or in education	445 (49.2)
Yes – currently employed or in education	460 (50.8)
Receiving Disability Living Allowance	
No	774 (85.5)
Yes	120 (13.3)
Prefer not to answer	11 (1.2)
Time off work in past 30 days (*n*, % of those in work)	
No	423 (92.0)
Yes	37 (8.0)
< 1 day	11 (2.4)
1 day	7 (1.5)
2–3 days	10 (2.2)
4–5 days	4 (0.9)
> 5 days	5 (1.1)
DLQI score for no time off work (*n* = 423)	
Mean (SD)	6.4 (5.9)
Median	5.0
Range	0–26
DLQI score for time off work (*n* = 37)	
Mean (SD)	11.8 (7.8)
Median	12.0
Range	0–26

*DLQI* Dermatology Life Quality Index, SD, standard deviation

## Discussion

In all conditions, the importance of obtaining patients’ views to assess the impact of the disease is becoming increasingly recognised. However, there is little published information about the treatment, management, and impact of psoriasis on patients in the United Kingdom from the patients’ perspective.

This survey was designed primarily to gain more insight on the impact of psoriasis on patients in primary care in the UK (using a patient cohort from the West of Scotland) by evaluating DLQI scores. The DLQI is the most widely used tool to measure the QoL impact of psoriasis. Although clinicians may undertake an “objective” assessment of disease severity, the significant psychosocial impact associated with limited disease may go unrecognised without the use of the DLQI (or similar instruments) to measure the impact of psoriasis on a patient’s QoL. The SIGN guidelines emphasise the importance of using the DLQI in primary care to assess the impact of the condition and to guide appropriate specialist referral.

The correlation of DLQI with PASI/SAPASI in patients with psoriasis is not always simple; a previous study investigating the relationship between SAPASI and DLQI found a poor correlation between the two measures [8] And although our data showed a reasonable correlation, there was considerable variability (Fig. 3). Limited disease at difficult, sensitive sites such as nails and genitals may lead to an imbalance between disease severity assessed by PASI/SAPASI and the impact of the disease assessed by the DLQI. Hence, we have focussed on using the DLQI to assess the impact of the condition in our analyses.

The SIGN guidelines recommend referral to a specialist for patients with psoriasis treated by a GP who do not respond to topical therapy and have a DLQI >5. In this cross-sectional survey, 65.5 % of those with a DLQI >5 had not seen a specialist during the past year, suggesting that a significant proportion of patients are not being referred to specialists despite SIGN guideline recommendations.

This study also highlighted an unmet need for more effective treatment, as a significant proportion of respondents had a DLQI >10, indicating that psoriasis had a very large or extremely large effect on their lives, were receiving no treatment (7 %), or only topical treatment (22 %). A recent, self-administered postal questionnaire conducted with 1564 members of the Psoriasis Association also showed that patients with psoriasis generally were dissatisfied with their current treatment [9].

Our survey population was comparable to the general population regarding the number of patients who smoked and had depression/anxiety; 23.4 % of our survey population reported being smokers compared with 20 % for those aged older than 16 in the UK and 16.8 % reported depression/anxiety compared with 17.2 % of those aged older than 16 having some evidence of depression/anxiety in Scotland [10, 11]. However, other comorbidities associated with severe disease, such as cardiovascular disorders, were reported more commonly in our population than in the general population (27.3 vs 10.1 % of the population older than 16 years with a long-standing condition of the heart and circulatory system according to the Office of National Statistics) [12].

The PEST questionnaire revealed that although 29 % of patients self-reported symptoms suggestive of psoriatic arthritis (indicating the need for referral to a rheumatologist), a considerably smaller proportion reported a diagnosis of the condition or had had it recorded in their medical records. There was good agreement between patient reporting and GP medical records (after secondary review of the GP records) relating to the diagnosis of psoriatic arthritis, suggesting that patient recollection of the diagnosis was excellent. It is also of some concern that the initial reports obtained from the GP survey were incomplete. GPs generally seemed to underrecord psoriatic arthritis. It is a condition that often goes undiagnosed in primary care, possibly due to factors such as lack of awareness, time constraints, or symptoms attributed to an alternative diagnosis. Of those reported with the condition by a GP, most correlated with patient-reported PEST, although some did not, possibly due to confusion with other types of arthritis.

Although psoriasis affects as much as 2 % of the UK population, most of whom are managed in primary care, there is little detailed information in the literature regarding psoriasis management in this setting from a GP and patient perspective [13]. The fact that many patients feel that their GP lacks specialist knowledge of the condition and the available treatments suggests a need for improved GP training, and for an increase in the number of dermatology specialist nurses in primary care settings. Qualitative studies, such as that conducted by Nelson and co-workers, have highlighted the need for GPs to improve their skills in the management of psoriasis and for long-term initiatives in partnership with patients that focus on improving QoL [9, 13]. The combination of qualitative and individual case analysis with quantitative assessments of QoL could make a valuable impact on future research into psoriasis and the development of suitable treatments [14].

Our study has several limitations, including its cross-sectional methodology. Medical history was self-reported and may be subject to recall bias. Although there was no selection bias in the practices approached to participate in the study, there will have been some self-selection in practices and patients willing to participate in the survey. Weight and height data to verify self-reported obesity was lacking.

Strengths of the study include the large sample size from patients in the primary care setting and a high completion rate for all questions. Medical records were reviewed for 76 % of the survey participants, which allowed correlation of data collected from both sources.

## Conclusions

This study showed that from a patient perspective, the burden of illness from psoriasis was significant and that patient reporting of the diagnosis of psoriatic arthritis was accurate, particularly where a specialist was involved in the diagnosis. There was evidence that SIGN guideline recommendations are not being fully implemented in primary care for patients with psoriasis or psoriatic arthritis, possibly due to time constraints or a lack of awareness. Further studies are warranted to determine the reasons for the lack of adherence to SIGN guidance and to make recommendations for improving adherence.

## Abbreviations

COPD: Chronic obstructive pulmonary disease
DLA: Disability living allowance
DLQI: Dermatology life quality index
GP: General practitioner
HCP: Healthcare provider
ICMJE: International Committee of Medical Journal Editors
NICE: National Institute for Health and Care Excellence
PASI: Psoriasis area and severity index
PEST: Psoriasis epidemiology screening tool
QoL: Quality of life
SAPASI: Self-administered psoriasis area and severity index
SD: Standard deviation
SIGN: Scottish intercollegiate guidelines network
UK: United Kingdom
